# Cardiac cycle-synchronized electrical muscle stimulator for lower limb training with the potential to reduce the heart's pumping workload

**DOI:** 10.1371/journal.pone.0187395

**Published:** 2017-11-08

**Authors:** Ken-ichiro Sasaki, Hiroo Matsuse, Ryuji Akimoto, Shiro Kamiya, Toshio Moritani, Motoki Sasaki, Yuta Ishizaki, Masanori Ohtsuka, Takaharu Nakayoshi, Takafumi Ueno, Naoto Shiba, Yoshihiro Fukumoto

**Affiliations:** 1 Division of Cardiovascular Medicine, Department of Internal Medicine, Kurume University School of Medicine, Kurume, Japan; 2 Department of Rehabilitation, Kurume University Hospital, Kurume, Japan; 3 Homerion Laboratory CO., LTD, Tokyo, Japan; 4 Laboratory of Applied Physiology, Kyoto Sangyo University, Kyoto, Japan; University of Debrecen, HUNGARY

## Abstract

**Background:**

The lower limb muscle may play an important role in decreasing the heart’s pumping workload. Aging and inactivity cause atrophy and weakness of the muscle, leading to a loss of the heart-assisting role. An electrical lower limb muscle stimulator can prevent atrophy and weakness more effectively than conventional resistance training; however, it has been reported to increase the heart’s pumping workload in some situations. Therefore, more effective tools should be developed.

**Methods:**

We newly developed a cardiac cycle-synchronized electrical lower limb muscle stimulator by combining a commercially available electrocardiogram monitor and belt electrode skeletal muscle electrical stimulator, making it possible to achieve strong and wide but not painful muscle contractions. Then, we tested the stimulator in 11 healthy volunteers to determine whether the special equipment enabled lower limb muscle training without harming the hemodynamics using plethysmography and a percutaneous cardiac output analyzer.

**Results:**

In 9 of 11 subjects, the stimulator generated diastolic augmentation waves on the dicrotic notches and end-diastolic pressure reduction waves on the plethysmogram waveforms of the brachial artery, showing analogous waveforms in the intra-aortic balloon pumping heart-assisting therapy. The heart rate, stroke volume, and cardiac output significantly increased during the stimulation. There was no change in the systolic or diastolic blood pressure during the stimulation.

**Conclusion:**

Cardiac cycle-synchronized electrical muscle stimulation for the lower limbs may enable muscle training without harmfully influencing the hemodynamics and with a potential to reduce the heart’s pumping workload, suggesting a promising tool for effectively treating both locomotor and cardiovascular disorders.

## Introduction

Aging leads to a loss of skeletal muscle mass and strength, specifically, sarcopenia and dynapenia, respectively [1,2,3]. They not only decrease the activities of daily life but also increase the risk of falling and fracturing bones in elderly people, resulting in frailty [4]. Chronic diseases, such as cardiovascular, pulmonary, diabetic, and malignant diseases, have been reported to occur with sarcopenia and dynapenia [1,2,5]. The chronic symptoms of the diseases may decrease the patient activity and precipitate them. The calf muscle may assist the heart’s pumping ability in a sitting or standing posture by pushing deoxygenated blood in the lower limbs up to the heart and by facilitating diastolic filling of the capillaries [6,7,8]. Accordingly, sarcopenia and dynapenia of the calf muscle may increase the heart’s workload and worsen the cardiovascular disease condition [6].

Heart failure is a global pandemic affecting more than 60 million patients in the United States, Europe, and Asia [9,10]. An estimated prevalence of frailty in patients with heart failure has been recently reported to be approximately 40–50% in a meta-analysis [11], suggesting that a promising tool for effectively treating both locomotor and cardiovascular disorders is necessary. Muscular strength has been reported to have an independent protective effect on all-cause mortality in men with hypertension and patients with heart failure [12] and coronary artery disease [13]. Additionally, resistance training for lower limbs has been reported to improve the exercise capacity and prognosis in patients with cardiovascular disease [14,15,16]. Thus, resistance training is recommended for preventing sarcopenia and dynapenia [17,18]; however, implementation using weights and machines often ends unsuccessfully in outpatients. Boring, repetitious, and stressful exercise programs may be a cause. In patients with orthopedic disease, such as osteoarthritis and lower back pain, the training methods must be challenging.

Electrical muscle stimulation (EMS), which contracts skeletal muscles via percutaneous electrodes that depolarize underlying motor nerves, has been reported as an alternative training method to prevent muscle atrophy and weakness for inactive patients [19,20,21]. The simplicity and passivity of the training method may allow outpatients to continue the training for a long time. However, there has been an unfavorable report that EMS to the lower limbs increases blood pressure and peripheral vascular resistance [22]. EMS-induced muscle contraction may increase the intramuscular pressure and elevate the peripheral vascular resistance. The elevation most likely increases the cardiac afterload and the heart’s pumping workload, resulting in undesirable hemodynamic changes such as stroke volume reduction. This notion cannot be discounted as a method to provide safe EMS therapy for patients with heart failure.

However, there has been an interesting report that EMS to the lower limbs decreases the vascular resistance when the muscle contraction is generated in a cardiac recovery phase [22]. If we are able to control EMS to the lower limbs to reduce the heart’s pumping workload based on cardiac cycle information, the EMS therapy may be safe for subjects and desirable for patients with heart failure. Recently, belt electrode skeletal muscle electrical stimulation (B-SES) has been reported as a new technology to allow for strong and wide but not painful skeletal muscle contractions [23]. Stronger and wider contractions of the lower limb muscle by B-SES compared with conventional EMS may augment the effect of reducing the heart’s pumping workload.

Accordingly, in order to test the hypothesis, we incorporated a special hand-made electrocardiogram signal regulation system into the conventional belt electrode skeletal muscle electrical stimulator and tried to achieve cardiac cycle-synchronized electrical skeletal muscle contraction of the lower limbs and aimed to examine whether the muscle contraction reduced the heart’s pumping workload in healthy subjects.

## Materials and methods

### Subjects

Eleven healthy male volunteers, who gave written informed consent, participated in this study. Subjects without any diseases and atherosclerotic risk factors such as current smoking, hypertension, dyslipidemia, and diabetes were recruited by a public offering at Kurume University Hospital. This study conformed to the principles outlined in the Declaration of Helsinki and was approved by the Committees on the Ethics Review Board of the Kurume University School of Medicine.

### Cardiac cycle-synchronized B-SES experimental protocol

**Fig 1:**
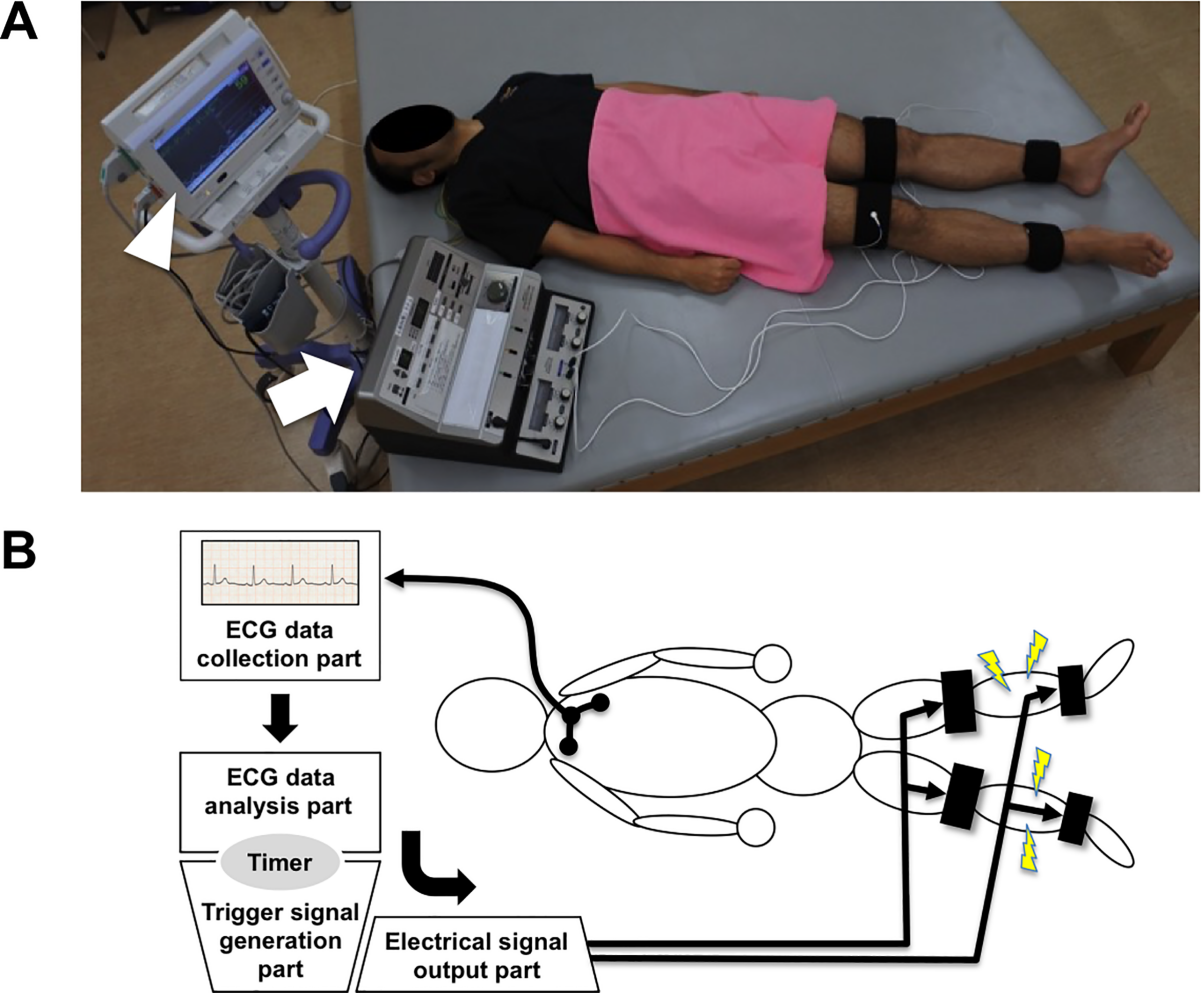
Cardiac-cycle synchronized belt electrode skeletal muscle electrical stimulation (C-B-SES) system for humans. (A) Four belt electrodes connected to the special device (white arrow) were wrapped around bilateral distal parts of the thigh and crus of the subject. The special device was linked to the electrocardiogram (ECG) recorder device (white arrowhead) through the special code. Written informed consent was obtained from the subject for publication of this picture. (B) Three electrodes pasted on the precordia of the subject were connected to the ECG recorder device to collect the ECG data. After collecting the data, they were sent to the special device and then C-B-SES was provided to the lower limbs. The special device combined the ECG data analysis, trigger signal generation, and electrical signal output for C-B-SES.

Special cloth-covered silicon rubber belt electrodes were attached on Velcro straps and then wetted with tepid water. The belt electrodes were wrapped around bilateral distal parts of the thigh and crus (Fig 1A) and were connected to a special device (white arrow in Fig 1A). Three electrocardiogram electrodes were pasted on the precordia (Fig 1B) and connected to an electrocardiogram (ECG) recorder device (Fig 1B and white arrowhead in Fig 1A; DYNASCOPE DS-8100 system, Fukuda Denshi, Japan). The two devices were linked through a special code.

**Fig 2:**
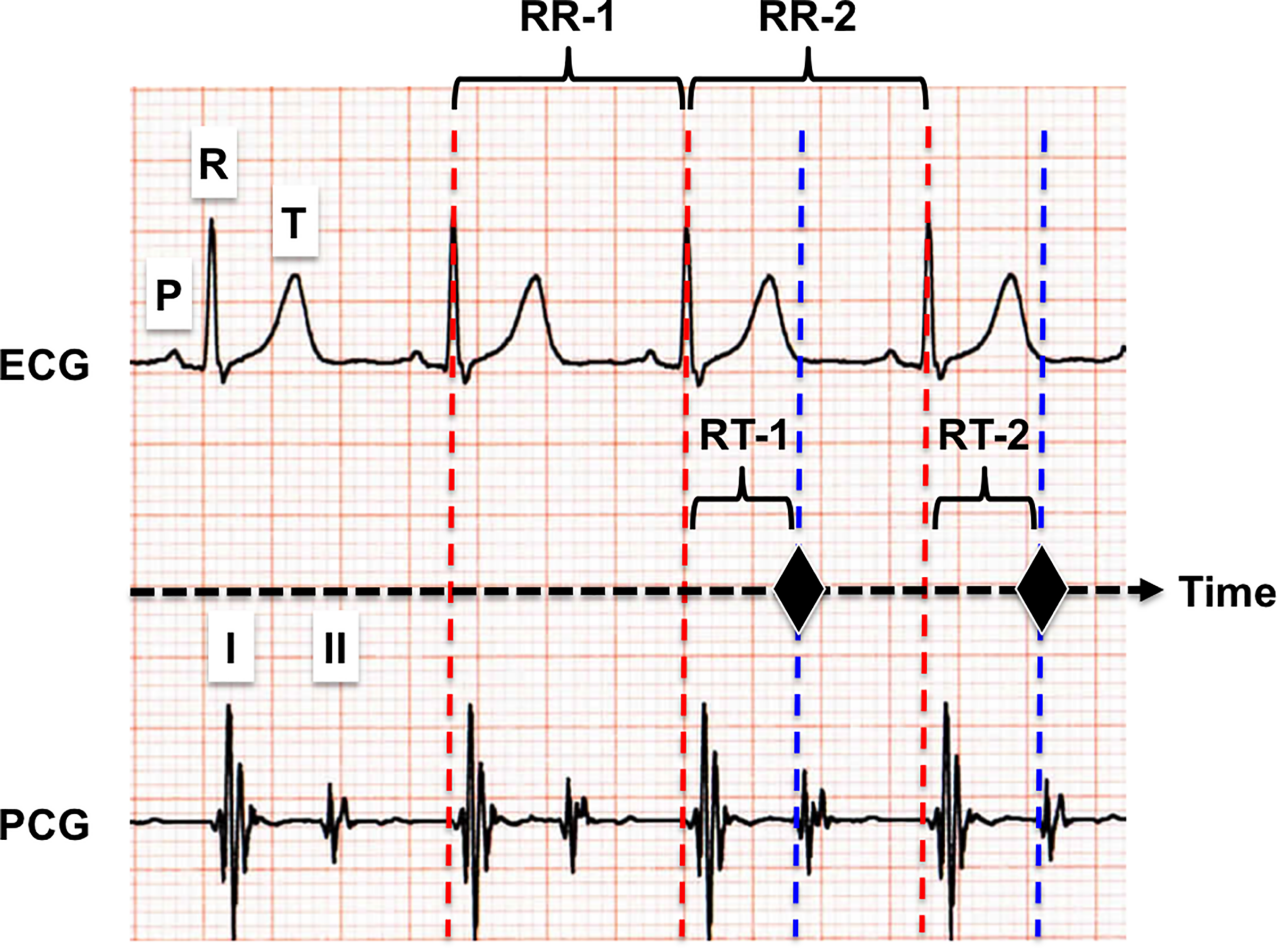
Synchro-time of C-B-SES on electrocardiogram (ECG) and phonocardiogram (PCG). P, R, and T indicate the P wave, R wave, and T wave of the ECG, respectively. I and II indicate the sound of the closing of the mitral and aortic valves, respectively. RR-1 and RR-2 indicate the interval time between the tops of the two R waves. RT-1 and RT-2 indicate the trigger signal generation time between the top of the R wave (red dashed line) and the synchro-time (blue dashed line under the black diamond). The black diamond indicates the time phase of C-B-SES on the ECG and PCG charts.

The cardiac cycle-synchronized B-SES (C-B-SES) system comprised the following four parts: ECG data collection; ECG data analysis; trigger signal generation; and electrical signal output (Fig 1B). The C-B-SES was controlled by the trigger signal. The ECG data collection in the ECG recorder device detected ECG waveforms of the subject and collected the ECG data. The ECG recorder device output the data for the next special device including the other three parts. The ECG data analysis part measured the R-R interval time (RR-1 and RR-2 in Fig 2) and calculated the trigger signal generation time (RT-1 and RT-2 in Fig 2) by multiplying the R-R interval time by a prescribed value that was adopted in order from 0.05, 0.075, 0.1, 0.15, 0.2, 0.25, 0.3, 0.35, 0.4, and 0.45. The calculated time is depicted below as ‘synchro-time’. The trigger signal (black diamonds in Fig 2) was generated after a delay of the synchro-time behind the last R-wave peak time phase (red and blue dashed lines in Fig 2). A timer in the special device (Fig 1B) regulated the synchro-time. When the electrical signal output part received the trigger signal, the lower limb muscle was electrically stimulated via the belt electrodes on the limb (Fig 1B).

For the experiment, we set the electrical stimulus frequency, pulse width, and duration to 20 Hz, 260 μsec, and 200 msec, respectively. The stimulus peak intensity was adopted between 56 and 106 mA according to individual resistance. One minute after the B-SES was controlled by a trigger signal, we checked whether the adopted synchro-time was suitable for cardiac cycle-synchronized electrical muscle stimulation with a reduction in the heart’s pumping workload using plethysmography (VaSela VS-3000, Fukuda Denshi, Japan), enabling ECG recording, phonocardiogram (PCG), and plethysmogram (PTG) together. If the recorded wave pattern was comparable with the pattern of IABP, the adopted synchro-time was judged to be suitable. In addition, we simultaneously measured a real-time stroke volume and cardiac output using a percutaneous analyzer (AESCULON mini^®^, Osypka Medical, Berlin, Germany). The instantaneous peripheral vascular resistance was calculated as the ratio of mean blood pressure to stroke volume [22].

The above numerical values were adopted to achieve the strongest skeletal muscle contraction without pain on the basis of previous reports [14,18,19,20] and preliminary experiments (data not shown).

### Statistical analysis

Continuous variables are presented as the mean ± SD. Statistical comparisons were performed using a nonparametric paired t-test. Statistical significance was assumed at a value of p<0.05. Data were analyzed using JMP Pro 11.0 (SAS Institute, Cary, NC, USA).

## Results

**Fig 3:**
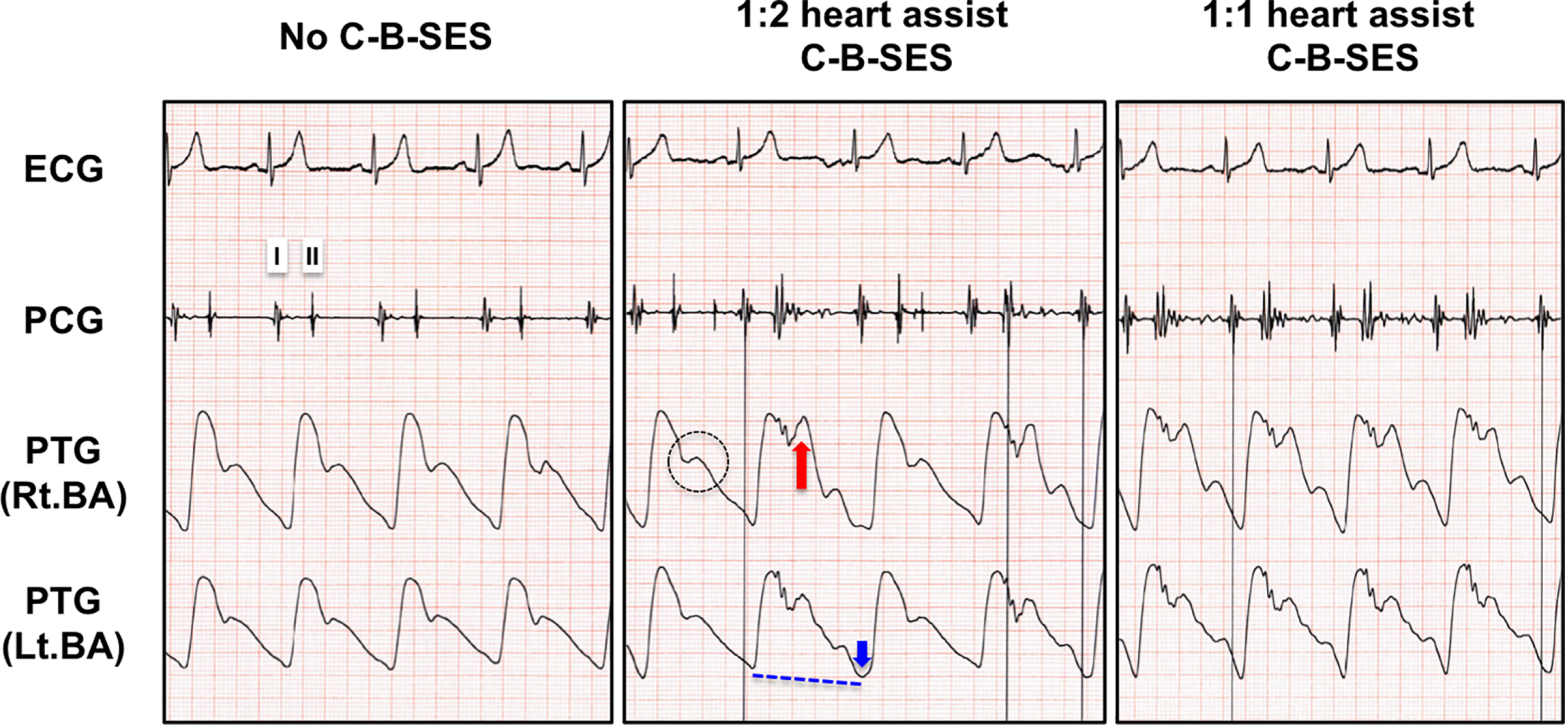
Representative plethysmography waveform charts during C-B-SES with heart assist effects. The chart displays ECG, PCG, and plethysmogram (PTG) together. The PTG waveforms are derived from bilateral brachial artery (BA) pulsation. I and II indicate the sound of the closing of the mitral and aortic valves, respectively. The black dashed line circle indicates a dicrotic notch corresponding to the acute drop following the systolic peak of the arterial pressure pulse wave. The notch coincides with the aortic valve closure. The red arrow indicates a diastolic augmentation wave on the dicrotic notch. The blue arrow indicates an end-diastolic pressure reduction wave. The end-diastolic pressure following C-B-SES was lower than the preceding unassisted end-diastolic pressure (blue dashed line).

**Fig 4:**
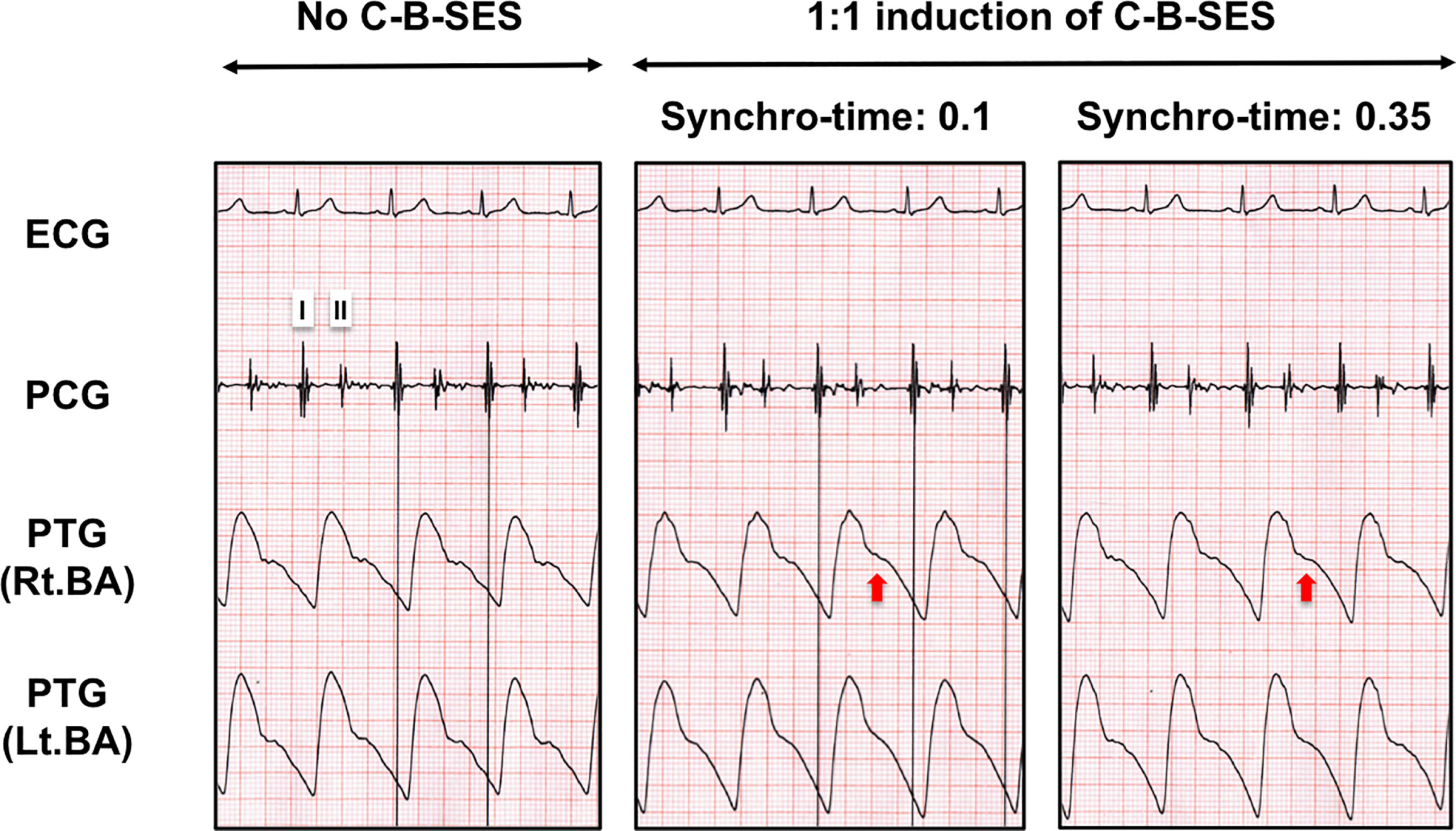
Representative plethysmography waveform charts during C-B-SES without desirable heart assist effects. Although we tested a 1:1 heart assist C-B-SES in each synchro-time adopted in order from 10 values between 0.05 and 0.45, desirable diastolic augmentation waves on the dicrotic notches or end-diastolic pressure reduction waves were not observed in all values (the center and right panels show the charts at 0.1 and 0.35 synchro-times, respectively). Despite the small change, there might be diastolic augmentation waves on the dicrotic notches (red arrows).

**Fig 5:**
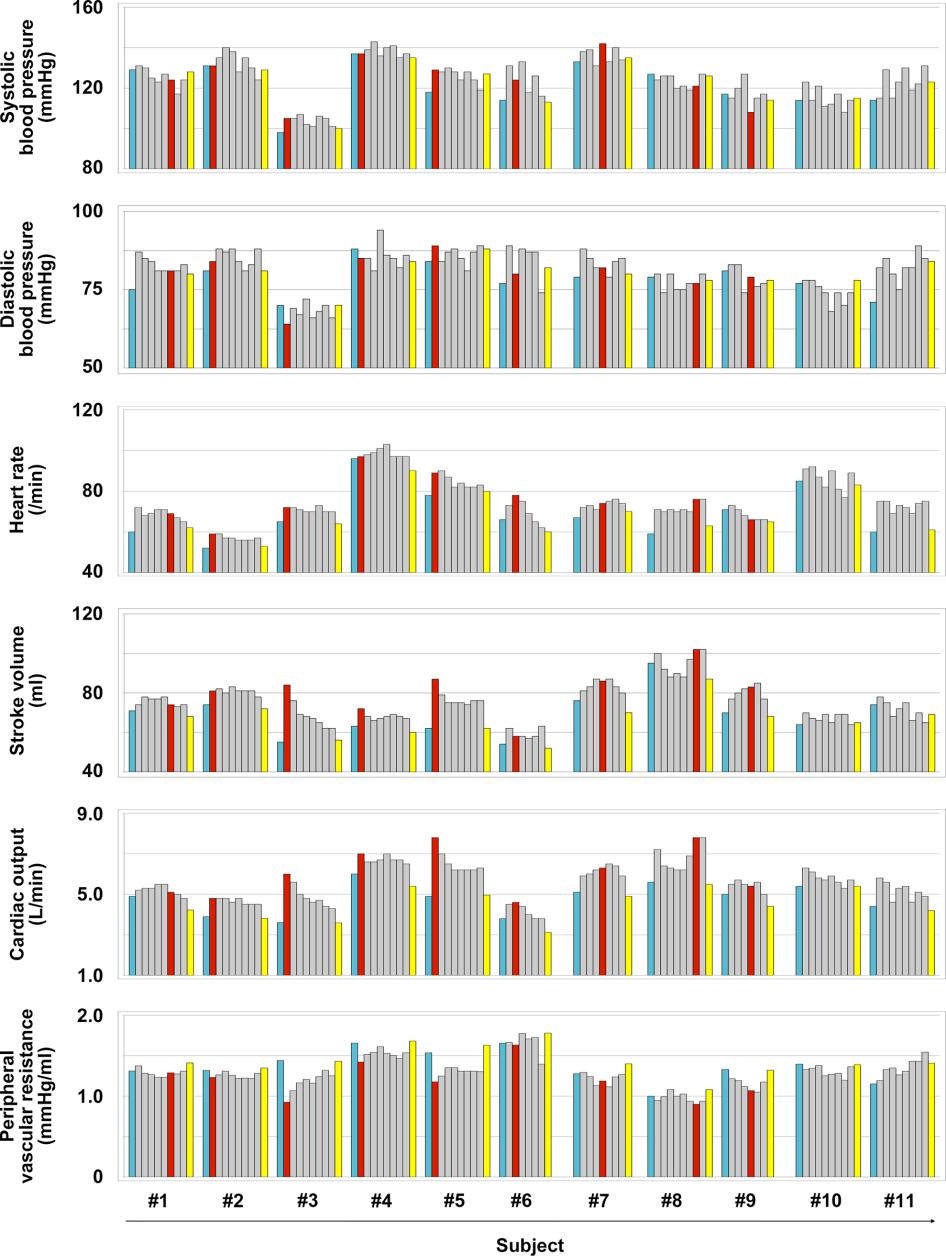
Trends of hemodynamic changes in the absence or presence of C-B-SES to the lower limbs. The blood pressure was measured by the plethysmography. The heart rate, stroke volume, and cardiac output were measured by the percutaneous analyzer. The peripheral vascular resistance was calculated using the value of the blood pressure and stroke volume. Trends in 11 subjects are shown by a bar-graphic representation for each subject. Each value expressed by a gray or red bar graph was the value in testing whether each adopted synchro-time was suitable for ideal C-B-SES. The red bar graph indicates the value when we achieved an ideal C-B-SES whose plethysmography wave pattern was comparable with the pattern of IABP. The blue and yellow bar graphs indicate the values before and after the test, respectively. The ideal C-B-SES was not achieved in subjects #10 and #11. The values of blue and red bar graphs correspond to the values of ‘OFF’ and ‘ON’ in Table 1, respectively.

In 11 participants in this study, the mean age, height, body weight, and body mass index were 38.9±7.2 years, 172.3±7.9 cm, 68.8±7.2 kg, and 23.1±0.9 kg/m^2^, respectively. Fig 3 shows representative plethysmography charts in the presence or absence of a favorable C-B-SES to the lower limbs. In a 1:2 heart assist C-B-SES (the center panel of Fig 3), a diastolic augmentation wave (red arrow in Fig 3) on a dicrotic notch (black dashed line circle in Fig 3) and an end-diastolic pressure reduction wave (blue arrow in Fig 3) appeared in the plethysmogram waveforms alternately. The augmented diastolic pressure during C-B-SES did not exceed the previous non-augmented systolic pressure, while the end-diastolic pressure following C-B-SES was lower than the preceding unassisted end-diastolic pressure (blue dashed line in Fig 3). The systolic pressure following a cycle of C-B-SES was not lower than the previous unassisted systolic pressure. In a 1:1 mode C-B-SES (the right panel of Fig 3), two such waves appeared in every waveform. These results were observed in 9 out of 11 subjects, but not in 2 others (Fig 4). The suitable synchro-times were 0.075 in 2 subjects, 0.1 in 4 subjects, 0.15 in 1 subject, 0.25 in 1 subject, and 0.35 in 1 subject. The heart rate (HR), stroke volume, and cardiac output significantly increased during C-B-SES that was controlled by the suitable synchro-time. The peripheral vascular resistance significantly decreased during the C-B-SES. There was no change in the systolic or diastolic blood pressure (Table 1). No subjects complained during or after the experiments. No complications for skin or muscle were observed. Even though an adopted synchro-time was not judged to be suitable, C-B-SES to the lower limbs occasionally increased cardiac output and/or decreased instantaneous peripheral vascular resistance (Fig 5).

**Table 1:** Hemodynamic changes in the absence or presence of C-B-SES controlled by the suitable synchro-time.

		C-B-SES (n = 9)		
		OFF	ON	
**SBP**	**(mmHg)**	**121.7±10.6**	**122.8±11.6**	**N.S.**
**DBP**	**(mmHg)**	**79.0±4.9**	**79.8±7.7**	**N.S.**
**HR**	**(/min)**	**68.2±12.8**	**75.6±11.6**	**p<0.01**
**SV**	**(ml)**	**70.0±13.2**	**80.9±11.1**	**p<0.005**
**CO**	**(L/min)**	**4.8±0.8**	**6.1±1.0**	**p<0.005**
**PVR**	**(mmHg/ml)**	**1.39±0.2**	**1.20±0.2**	**p<0.05**

SBP: systolic blood pressure; DBP: diastolic blood pressure; HR: heart rate; SV: stroke volume; CO: cardiac output; PVR: peripheral vascular resistance.

## Discussion

In this study, we developed new cardiac cycle-synchronized B-SES equipment for lower limbs and successfully achieved cardiac cycle-synchronized muscle contractions of the lower limbs in healthy subjects. Moreover, in suitable electrical stimulation, the muscle contraction seemed to reduce the heart’s pumping workload and increase the cardiac output. The plethysmography waveforms in achieving the effects were composed of a diastolic augmentation wave on a dicrotic notch and an end-diastolic pressure reduction wave, resembling the waveforms during intra-aortic balloon pumping (IABP), the most widely used strategy for achieving mechanical circulatory support.

The heart’s pumping ability is an important factor in regulating homeostasis of the blood circulatory system. In a sitting or standing posture, the residual heart’s pumping ability in the venous phase may be insufficient for pushing the deoxygenated blood up to the heart because the blood volume corresponds to ≈70% of all of the blood. The calf muscle is often called “the second heart” and is considered a candidate to help venous blood pumping. Aging and daily inactivity-induced sarcopenia and dynapenia for the calf muscle may bring a loss of the second heart’s assistance and worsen the disease condition, especially in patients with cardiovascular diseases. B-SES, which can contract the quadriceps femoris, hamstring, tibialis anterior, and triceps surae muscles simultaneously, has been reported to increase the muscular thickness and strength of lower limbs effectively by the strong and wide contraction ability of the muscles [23,24]. It may be that B-SES to the lower limbs prevents muscle atrophy and thereby maintains the second heart’s assistance. Moreover, B-SES training has been reported to improve glycometabolism and cardiorespiratory fitness [25,26], indicating a promising resistance training method for cardiovascular and metabolic patients with sarcopenia and dynapenia.

**Fig 6:**
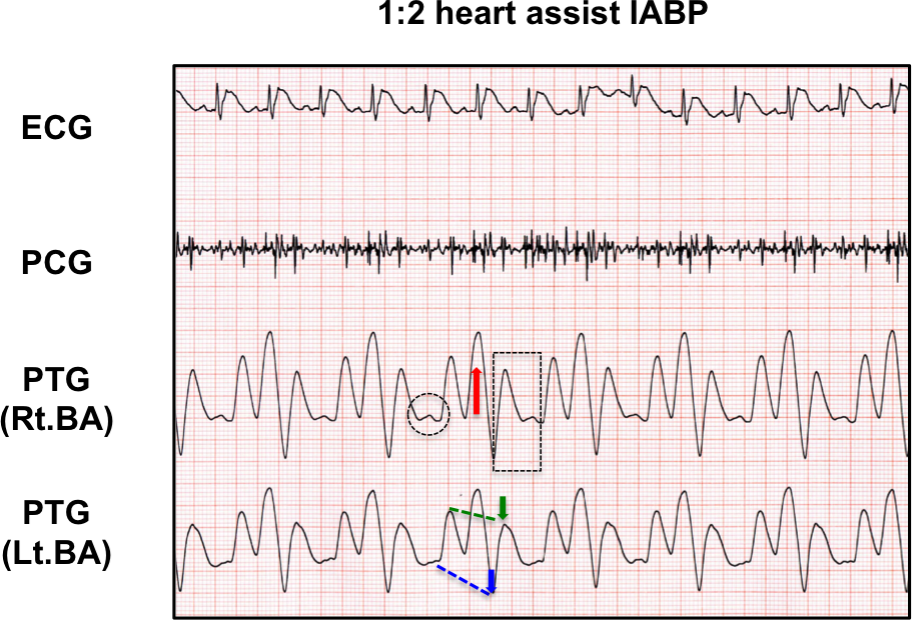
Representative plethysmography waveform chart in an ideal 1:2 heart assist IABP. The plethysmogram (PTG) waveforms are derived from bilateral brachial artery (BA) pulsation. The red arrow indicates a diastolic augmentation wave on the dicrotic notch (black dashed line circle). The blue arrow indicates an end-diastolic pressure reduction wave. The end-diastolic pressure following IABP was lower than the preceding unassisted end-diastolic pressure (blue dashed line). In addition, the systolic pressure following IABP was lower than the previous unassisted systolic pressure (green arrow and dashed line).

However, B-SES to the lower limbs may increase the heart’s pumping workload in some instances as reported in the application of conventional EMS [22]. To resolve the problems, we tried to develop a C-B-SES system by combining our specially modified B-SES equipment with a commercial electrocardiogram monitor. On the plethysmography waveform, C-B-SES to the bilateral lower limbs generated a diastolic augmentation wave on the diastolic notch and an end-diastolic pressure reduction wave of every muscle contraction in 9 of 11 subjects. Although the waves were similar to those seen in IABP (Fig 6), the diastolic augmentation wave height was not higher than the previous non-augmented systolic blood pressure wave height. An ideally assisted systolic blood pressure wave, of which height should be lower than the previously unassisted systolic blood pressure wave heights, was not seen in all subjects. A stronger electrical stimulation may bring ideal wave generation to all subjects; however, such painful and uncomfortable stimulation must be unsuitable for future clinical use. Future studies based on other approaches are needed to improve the present prototype. Nevertheless, even though the effect leads to a reduction in the heart’s pumping workload, the present C-B-SES will at least prevent traditional EMS-induced enhancement of peripheral vascular resistance and subsequent increases in cardiac afterload [22]. Additionally, C-B-SES induced diastolic augmentation most likely indicates an increase in coronary artery flow, indicating success for treating angina pectoris.

IABP therapy is the most widely used and most affordable form of mechanical circulatory support; however, the method of implication and the subsequent complication are relatively invasive [27]. Outpatients cannot receive the therapy because of the intra-aortic indwelling catheterization, which is inconvenient for outpatients who need mechanical circulatory support routinely. Enhanced external counter pulsation (EECP) therapy, an alternative therapy to IABP, provides hemodynamic support to patients with cardiac diseases [28,29,30] by using not a catheter but a pants-shaped compressive cuff that is firmly wrapped around the lower limbs. The cuff behavior modulates blood flow of the lower limbs for increasing coronary perfusion pressure and decreasing cardiac afterload, mimicking the hemodynamic consequences of IABP. However, the cuff on the skin often leads to contact dermatitis [28,31,32], and the large-sized unit does not save space and is not portable, suggesting an invasive and user-unfriendly therapeutic instrument. C-B-SES to the lower limbs is non-invasive, space-saving, and user-friendly compared with IABP and EECP, suggesting a long-term implication for routine mechanical circulatory support for outpatients with angina pectoris and chronic heart failure. Even if the effect may be partial at present, C-B-SES to the lower limbs may enable substitution for IABP and EECP in the future.

In EECP, the cuff deflation decreases the peripheral vascular resistance, increasing arterial blood inflow into the periphery. Then, the cuff inflation widely compresses the blood vessel of the lower limbs, increasing the venous return from the limbs into the right ventricle. In conjunction, the cardiac stroke volume and cardiac output increase [29]. In this study, C-B-SES to the lower limbs increased stroke volume and cardiac output. The mechanism would be comparable with that of EECP, suggesting that we should pay attention to whether the increased venous return worsens the medical condition in patients with pulmonary hypertension and regurgitant valvular heart diseases despite no report regarding EECP.

C-B-SES to the lower limbs increased HR. Although the increase in HR is related to the increase in cardiac output, the increase in HR may increase the heart’s workload undesirably in some situations of treating ischemic heart disease and heart failure. Although the muscle contraction by B-SES is passive, the wide and strong muscle contraction may require more arterial blood supply for the muscle from the heart. However, this hypothesis has not been tested in this study. It has been reported that a passive stretch of muscle increases HR by 6±2 beats per minute [33]. Muscle mechano- and metabo-receptors in exercising have been reported to regulate cardiac autonomic nerve activity and lead to an increase in HR [34], suggesting a mechanism by which passive stretching of the lower limb muscle by C-B-SES increased HR by 7.3±2.1 beats per minute. Although the heart rate variation was not parallel to blood pressure variation, psychological stress by C-B-SES possibly augmented sympathetic activity and influenced the heart rate and blood pressure variation. The variation pattern of the heart rate or blood pressure did not correspond to that of the cardiac output or peripheral vascular resistance. In addition, even though an adopted synchro-time was not suitable, C-B-SES to the lower limbs occasionally increased cardiac output and/or decreased peripheral vascular resistance. The non-invasively hemodynamic analyses in this study may be inadequate to resolve the above issues, suggesting the necessity to estimate hemodynamic effects on C-B-SES to the lower limbs correctly by an examination using intravascular catheterization, which enables us to record blood pressure, heart rate, stroke volume, cardiac output, and vascular resistance simultaneously. It has been reported that the HR increase is attenuated by the use of β1-adrenergic blockade. C-B-SES to the lower limbs in patients with cardiovascular diseases may not increase HR if they take a β-blocker daily for medicating the underlying illness.

In this study, there are several limitations. First, we have not tested C-B-SES for healthy females or patients with leg edema; females often have a thicker subcutaneous fat layer than males. If higher intensity electrical stimulation is necessary for sufficient muscle contraction in the subjects, it will be unfavorable for general use. Second, we have not tested C-B-SES for patients with arrhythmias such as premature contractions or atrial fibrillation. The ECG data analysis part and trigger signal generation part may be unable to work desirably because the R-R interval is irregular. These problems should be resolved before clinical use. Third, the sample size might have been relatively small to confirm the results.

In conclusion, the C-B-SES that we developed in this study may treat locomotor and cardiovascular disorders together without any negative hemodynamic effects. Hereafter, it is necessary to verify whether a prolonged C-B-SES to the lower limbs in relatively elder subjects with sarcopenia and /or dynapenia can improve such disorders in a large population.
